# Crystal structure and characterization of a new one-dimensional copper(II) coordination polymer containing a 4-amino­benzoic acid ligand

**DOI:** 10.1107/S2056989024001336

**Published:** 2024-02-20

**Authors:** Alisha Gogia, Egor M. Novikov, Ilia A. Guzei, Marina S. Fonari, Tatiana V. Timofeeva

**Affiliations:** a Department of Chemistry, New Mexico Highlands University, Las Vegas, New, Mexico, 87701, USA; b Chemistry Department, University of Wisconsin-Madison, 1101 University Ave, Madison, WI 53706, USA; c Institute of Applied Physics, Moldova State University, Academy str., 5 MD2028, Chisinau, Moldova; University of Durham, United Kingdom

**Keywords:** 4-amino­benzoic acid, *p*ABAH, one-dimensional coordination polymer, crystal structure

## Abstract

A Cu^II^ coordination polymer, *catena*-poly[[[aqua­copper(II)]-bis­(μ-4-amino­benz­o­ato)-κ^2^ *N*:*O*;κ^2^ *O*:*N*] monohydrate], {[Cu(*p*ABA)_2_(H_2_O)]·H_2_O}_*n*_ (*p*ABA = *p*-amino­benzoate, C_7_H_4_NO_2_^−^), was synthesized and characterized. It exhibits a one-dimensional chain structure extended into a three-dimensional supra­molecular assembly through hydrogen bonds and π–π inter­actions. While the twinned crystal shows a metrically ortho­rhom­bic lattice and an apparent space group *Pbcm*, the true symmetry is monoclinic (space group *P*2/*c*), with disordered Cu atoms and mixed roles of water mol­ecules (aqua ligand/crystallization water). The luminescence spectrum of the complex shows an emission at 345 nm, *cf.* 349 nm for *p*ABAH.

## Abstract 2

A new coordination polymer based on Cu^II^ and 4-amino­benzoic acid is isolated and characterized using single-crystal X-ray diffraction, FTIR and fluorescence spectroscopy, and thermal analysis.

## 1. Chemical context

**Figure 1:**
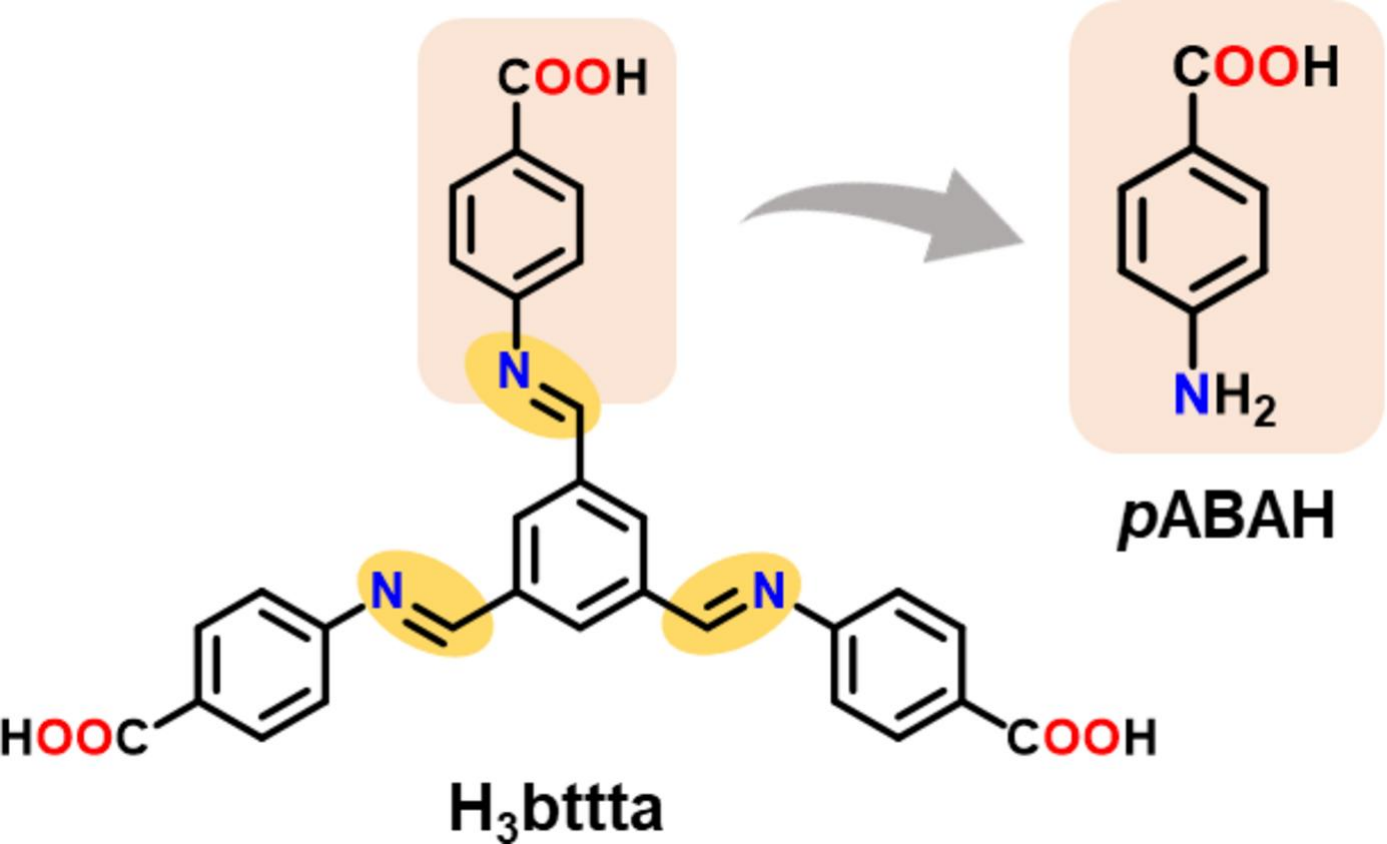
Tri­carb­oxy­lic ligand (H_3_btta) used and its fragmentation to *p*ABAH under hydro­thermal reaction conditions.

Coordination polymers (CPs), which can be categorized in the class of lower dimensional metal–organic frameworks (MOFs), have received great attention in the past few decades owing to the multitude of applications they offer, such as gas storage and separation (Férey, 2008 ▸), sensing (Horcajada *et al.*, 2012 ▸), drug delivery (Liu *et al.*, 2020 ▸), electrochemical applications (Morozan & Jaouen, 2012 ▸), adsorption and remedi­ation (Baruah, 2022 ▸), magnetic properties (Maspoch *et al.*, 2004 ▸), *etc*. Despite advancements, the anti­cipation of MOF structures remains an ongoing challenge. Even with reticular synthesis initiated by geometrically analogous ligands, the outcome of structures or ligand behaviors under elevated temperature and pressure conditions, prevalent during synthesis, remains complicated (Szczypiński *et al.*, 2021 ▸). Occasionally, in the pursuit of creating porous architectures, our efforts yield coordination polymers with unexpected features. In the present work, we attempted to synthesize a porous metal–organic framework based on Cu^II^ and a flexible tri­carb­oxy­lic acid ligand, 4,4′,4′′-{[(1*E*,1′*E*,1′′*E*)-benzene-1,3,5-triyltris(methane­ylyl­idene)] tris­(aza­neylyl­idene)}tri­benzoic acid (H_3_bttta) (Fig. 1 ▸). Instead, we obtained a one-dimensional CP, {[Cu(*p*ABA)_2_(H_2_O)]·H_2_O}_*n*_ (I), with the anion of *p*-amino­benzoic acid (*p*ABAH), the latter presumably formed by disintegration of H_3_bttta in the course of hydro­thermal synthesis. Subsequently we synthesized compound (I) from Cu(NO_3_)_2_·2.5H_2_O and *p*ABAH under the same synthetic conditions. Compound (I) was characterized by single-crystal X-ray diffraction, FTIR spectroscopy and thermogravimetric analysis (TGA). 
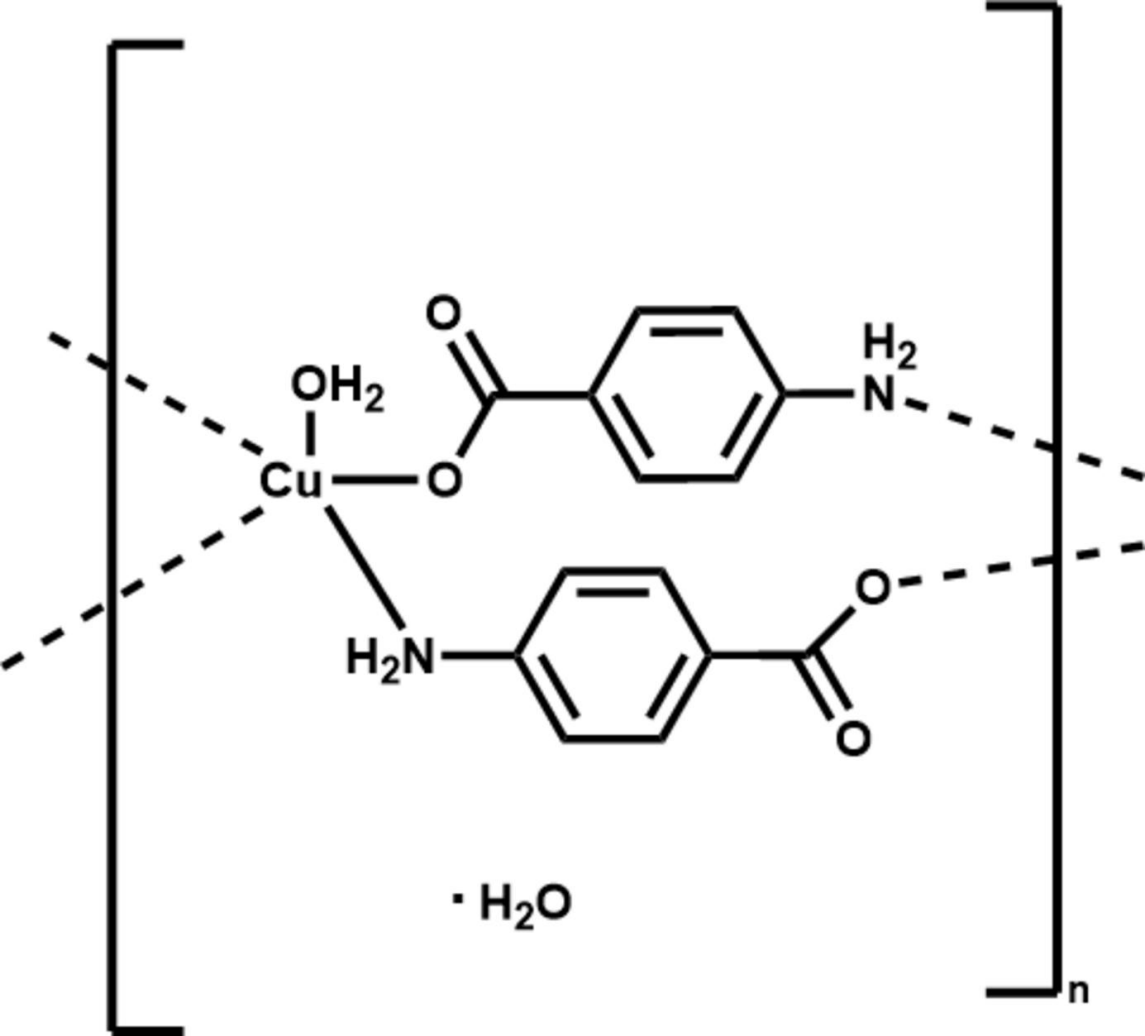


**Figure 2:**
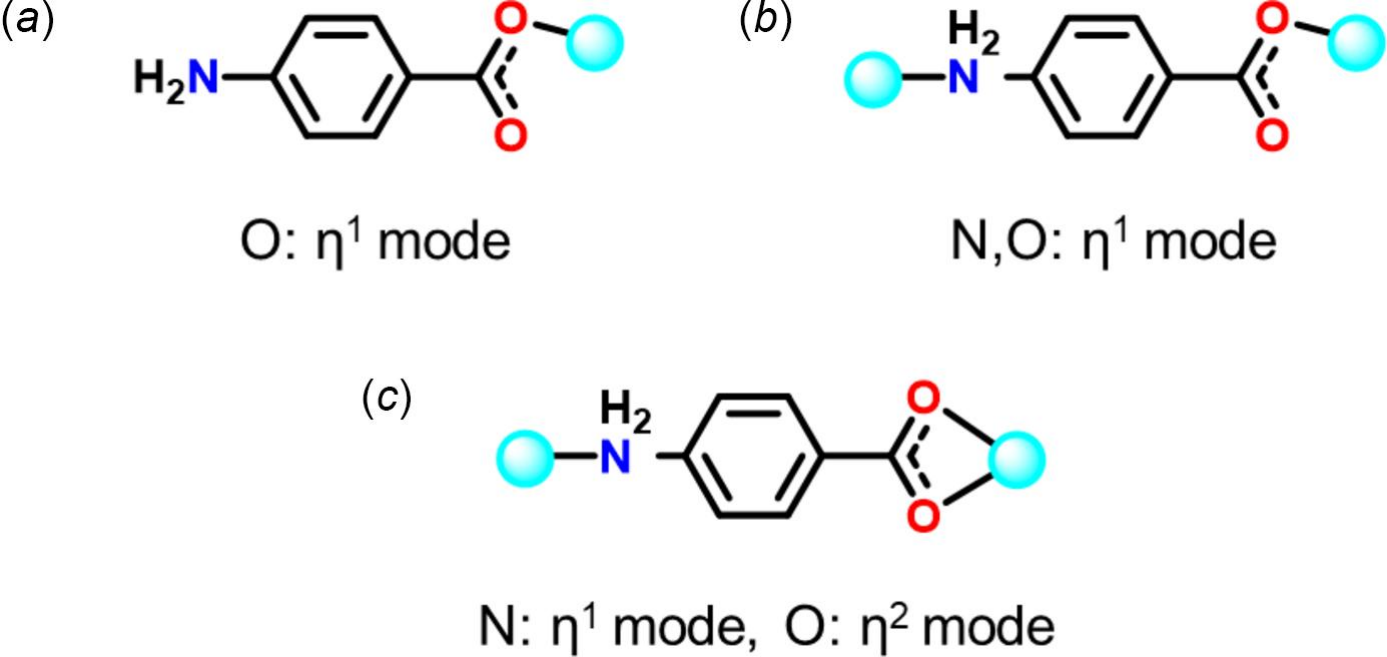
Binding modes of the *p*ABA ligand in coordination polymers with Co^II^, Ni^II^, Cu^II^, Zn^II^ or Cd^II^ (shown as blue spheres).

Its anion, *p*ABA, is capable of versatile binding with metal ions *via* amino and carb­oxy­lic groups (Fig. 2 ▸), as well as strong hydrogen bonds and π–π stacking inter­actions, enhancing the overall stability of the CP. Moreover, *p*ABAH has a variety of applications, *viz*. as precursor in the synthesis of pharmaceutical compounds, UV absorbers, components in hair dyes, anti­oxidants, food additives, *etc*.

## 2. Structural commentary

**Figure 3:**
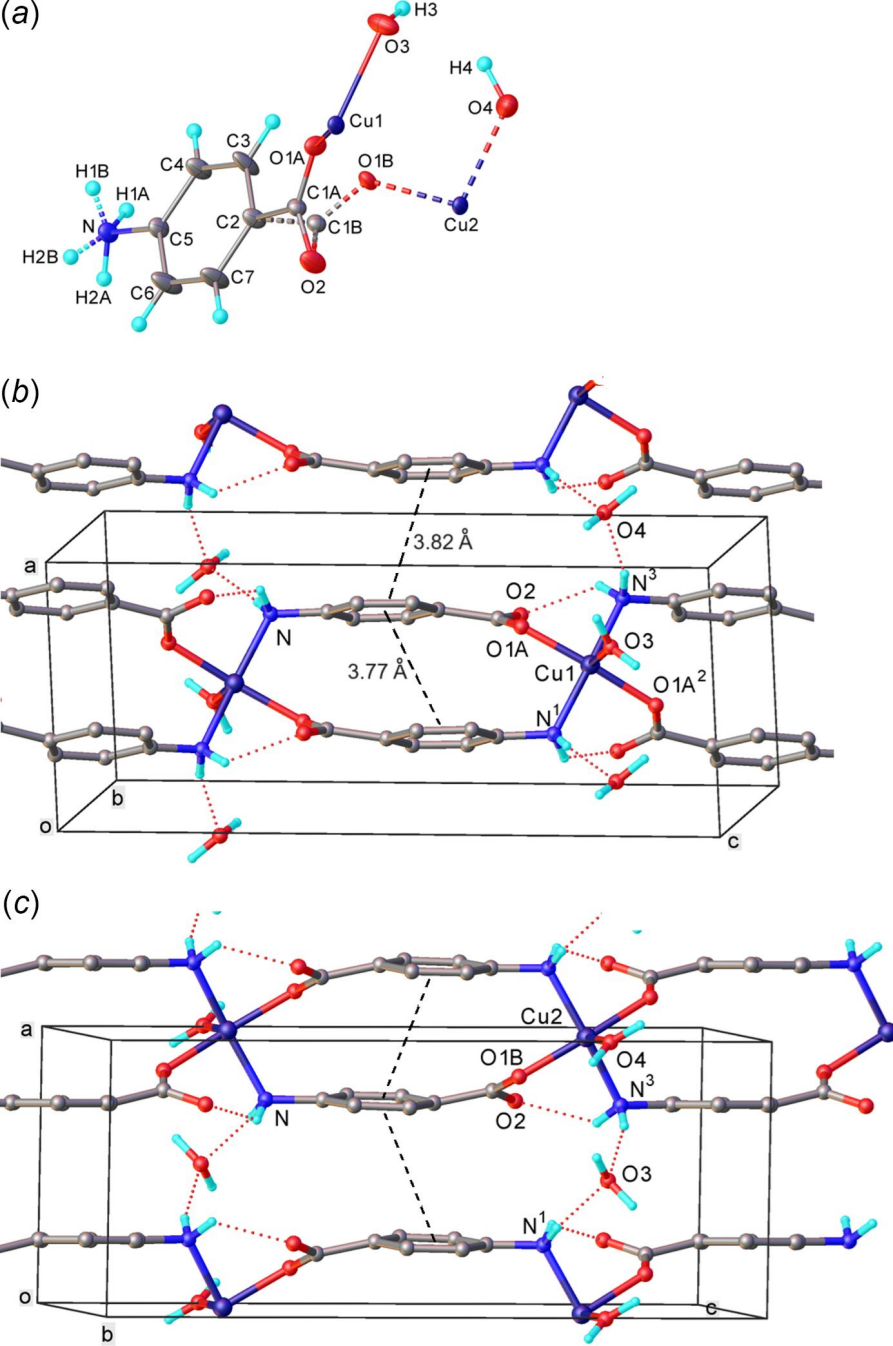
Disorder in the crystal of (I). (*a*) The asymmetric unit, showing atomic displacement ellipsoids at the 30% probability level. The major (solid) and minor (stippled) components have occupancies of 0.6196 (16) and 0.3804 (16), respectively. (*b*), (*c*) Crystal packing for these components. In the former, mol­ecule O4H_2_ acts as an aqua ligand, O3H_2_ as crystallization water, and *vice versa* in the latter. Hydrogen bonds are shown as dotted lines, π–π stacking as dashed lines between the centroids of arene rings. Symmetry codes: (1) 1 − *x*, 1 − *y*, 1 − *z*; (2) 1 − *x*, *y*, 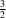 − *z*; (3) *x*, 1 − *y*, 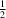 + *z*.

Compound (I) crystallizes in a monoclinic space group *P*2/*c*, although the unit cell is metrically ortho­rhom­bic. The asymmetric unit comprises half of Cu atom, one *p*ABA ligand and one water mol­ecule. The Cu atom is disordered between two alternative sites, Cu1 and Cu2, both located on crystallographic twofold axes, with crystallographic occupancies of 0.3098 (8) and 0.1902 (8), respectively. The carb­oxy­lic group is also disordered, the atomic sites C1*A* and O1*A* are occupied simultaneously with Cu1 and have occupancies of 0.6196 (16), whereas C1*B* and O1*B* are occupied simultaneously with Cu2 and have occupancies of 0.3804 (16). The H atoms of the amino group are also disordered between two sets of positions with the same occupancies, depending on whether the adjacent Cu1 or Cu2 site is occupied and coordinated with N1. The disorder is illustrated in Fig. 3 ▸.

It is noteworthy that the atomic positions (including those of the disordered atoms) approximately comply with the ortho­rhom­bic symmetry (apparent space group *Pbcm*), but their occupancies do not, therefore refinement of the structure in this symmetry gives a computationally unstable, as well as chemically and crystallographically unreasonable, model.

Both the Cu1 and Cu2 sites have an N_2_O_3_ square-pyramidal coordination environment, in which the apical position is occupied by an aqua ligand (*i.e.* the O3 or O4 atom, respectively), also located on a twofold axis. Note that the water sites, unlike the Cu ones, are fully occupied. Thus, if the Cu1 site is occupied and Cu2 is vacant, O3H_2_ is an aqua ligand and O4H_2_ is a water mol­ecule of crystallization and *vice versa* if the Cu2 site is occupied.

The *p*ABA ligand bridges two adjacent Cu atoms (related by the *c* glide plane) through amine nitro­gen and carboxyl­ate oxygen atoms in a μ_2_-*O*:*N* binding mode. Thus each Cu atom is linked with two symmetry-equivalent ones by pairs of anti­parallel *p*ABA ligands (whose two O and two N atoms comprise the basal plane of the pyramid), to form a polymeric chain parallel to the *c* axis.

## 3. Supra­molecular features

The one-dimensional *catena*-Cu(*p*ABA) chains of (I) are combined into a three-dimensional supra­molecular structure by a network of hydrogen bonds (Table 1 ▸). Both water mol­ecules (whether coordinated or not) donate hydrogen bonds to the non-coordinated carb­oxy­lic atom O2 (and its equivalents), forming an infinite zigzag chain O2⋯H—O3—H⋯O2⋯H—O4—H⋯O2 along the *a-*axis direction. The amino group, which is disordered over two orientations (see above), in either case donates one hydrogen bond to a trans-annular O2 and the other to the water mol­ecule, which is not coordinated (the adjacent Cu site being vacant). Thus, while an aqua ligand donates two hydrogen bonds, the crystallization water at the same site donates two and accepts two, from different adjacent Cu(*p*ABA) chains.

There is π–π stacking of practically parallel arene rings of *p*ABA (Fig. 3 ▸). Infinite stacks run parallel to the *a* axis, with alternating inter­planar separations of 3.41 (6) and 3.49  (6) Å, lateral shifts between adjacent rings of 1.72 (8) and 1.42 (9) Å, and distances between ring centroids of 3.82 (4) and 3.77 (4) Å, respectively.

## 4. Spectroscopic and thermal properties

**Figure 4:**
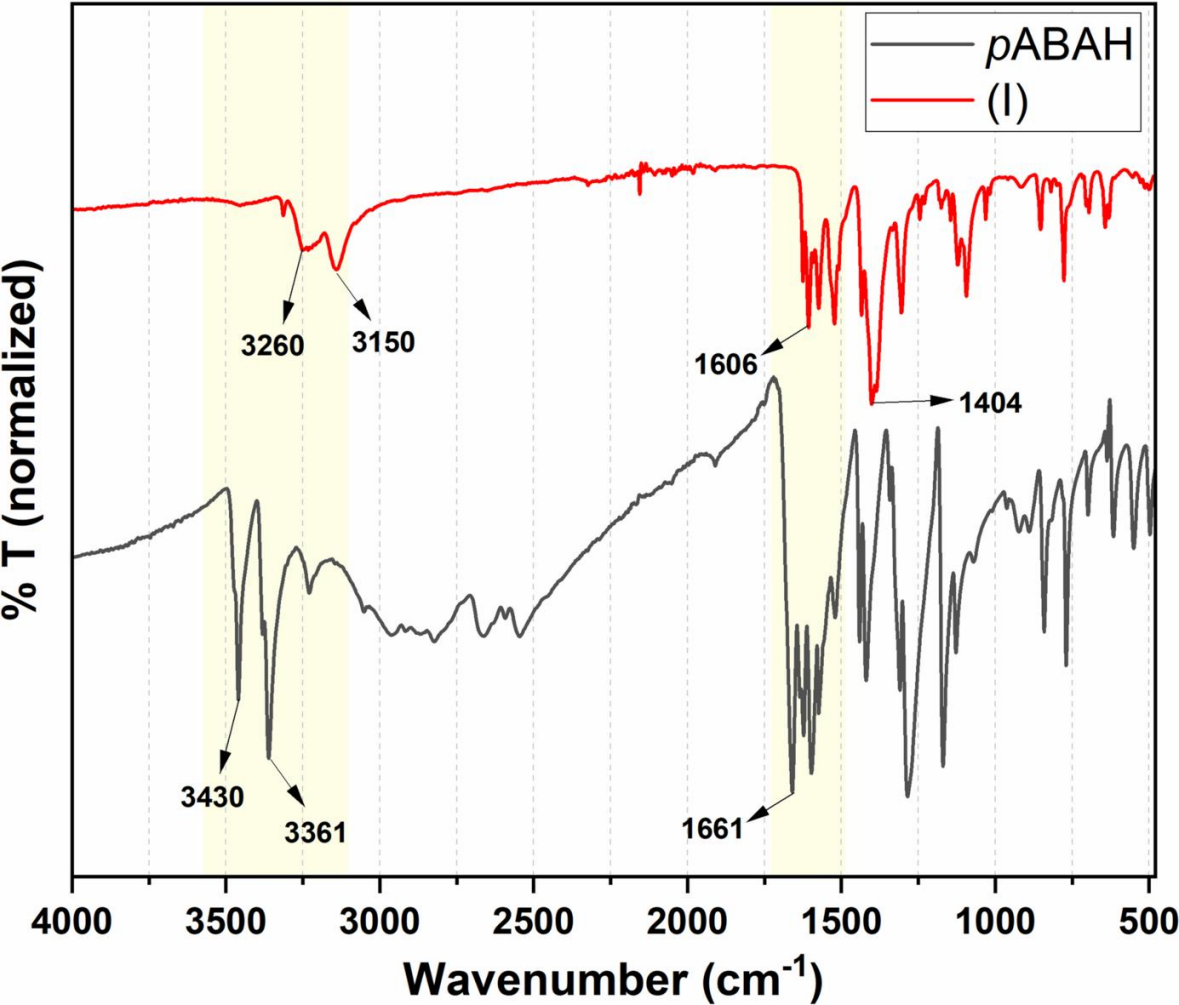
FTIR spectra of *p*ABAH (black) and compound (I) (red).

The FTIR spectra of *p*ABAH and (I) (Fig. 4 ▸) demonstrated successful incorporation of the *p*ABA ligand in (I). In comparison to the free ligand, *p*ABAH, the peaks corres­ponding to the amine group suffer a decrease in the wavenumber and intensity upon binding to the Cu^II^ atom in (I), similar to what is observed in other cases in the literature (Crisan *et al.*, 2019 ▸). In addition, the peak at 1661 cm^−1^, corresponding to the free carb­oxy­lic acid in *p*ABAH is diminished upon metal coordination in (I), Fig. 4 ▸. The strong bands at 1606 cm^−1^ and 1404 cm^−1^ correspond to the asymmetric (ν_asym_) and symmetric (ν_sym_) stretching vibrations of the carboxyl­ate group of *p*ABA in (I). The difference in the asymmetric and symmetric vibrations (Δν = 202 cm^−1^) corresponds to monodentate binding of the carboxyl­ate which corroborates well with the structure of (I).

**Figure 5:**
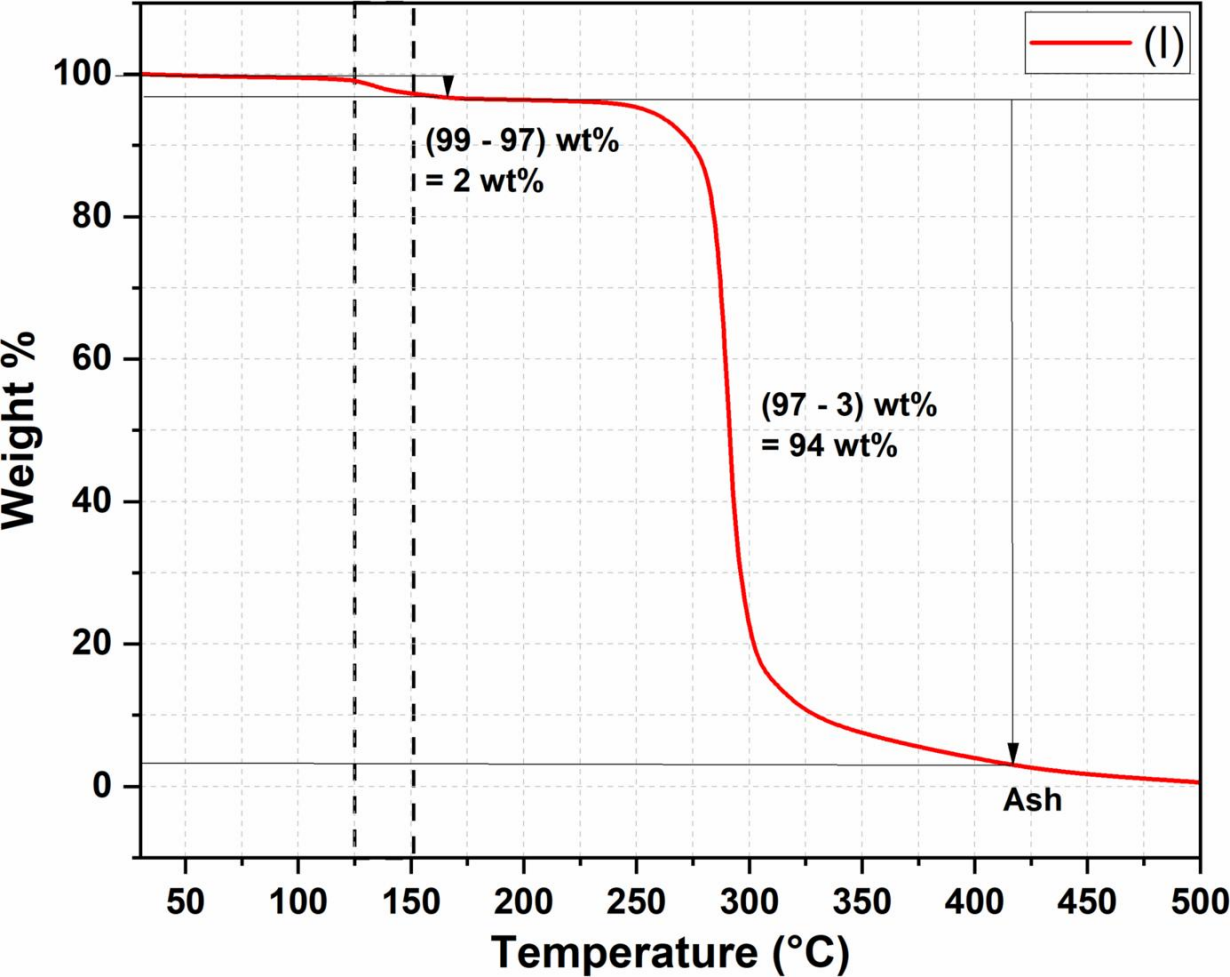
Thermogravimetric analysis of (I).

The stability of (I) was studied by thermal gravimetric analysis in the range of 30-500°C, which shows that (I) is stable up to 300°C. The initial loss of 2 wt% corresponds to the loss of coordinated water mol­ecules, and the complete decomposition (94 wt%) corresponds to the evolution of CO_2_ upon the decomposition of the carboxyl­ate group in the ligand, *p*ABA, leaving behind metal oxide ash (Fig. 5 ▸). The percentage of ash left behind is surprisingly lower than expected and might be due to the heterogeneity of the material.

## 5. Luminescence properties

**Figure 6:**
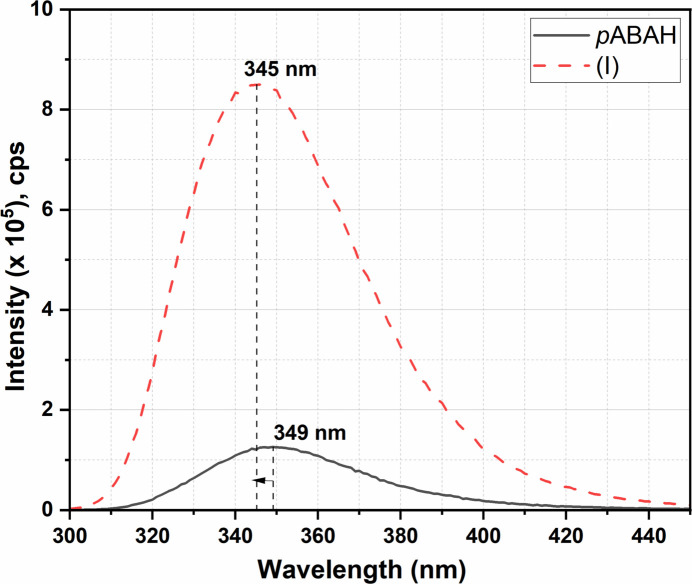
Luminescence emission spectra of pure *p*ABAH and (I) measured at room temperature in water (λ_excitation_ = 280 nm).

The emission spectra of (I) and the *p*ABA ligand were recorded at room temperature to assess the luminescence properties of the samples. For this, 1 mg of each sample was finely dispersed in 2 mL of water through ultrasonication. Their respective emission spectra were then recorded at an excitation wavelength of 280 nm, and excitation and emission slit widths of 1 and 1 nm, respectively, in the range 300 to 450 nm. It was found that the emission intensity of (I) is much more intense compared to the emission intensity of the pure *p*ABAH ligand in water. Compound (I) also undergoes a slight blue shift of Δλ = 4 nm, which is representative of the binding of ligand (*p*ABA) with the metal center (Cu^II^) (Fig. 6 ▸).

## 6. Database survey

Although *p*ABA is widely used as a ligand in the synthesis of coordination polymers and metal–organic frameworks, a survey of the Cambridge Structural Database (version 5.45, updated on 01/01/2024; Groom *et al.*, 2016 ▸) revealed no Cu complexes containing only *p*ABA ligands and coordinated or crystallization water, while such complexes are known for Co^II^, Ni^II^, Zn^II^ and Cd^II^. Most of these are one-dimensional coordination polymers, although [Co(*p*ABA)(H_2_O)_4_] (ABZACO10; Amiraslanov *et al.*, 1979*a* ▸) crystallizes as discrete mol­ecular units, [Zn(*p*ABA)_2_(H_2_O)]·H_2_O (IWORET; Ibragimov *et al.*, 2016 ▸) as a two-dimensional polymer, and [Zn(*p*ABA)_2_]·H_2_O (RUPZIM; Li *et al.*, 2009 ▸) as a three-dimensional MOF. The carb­oxy­lic group of *p*ABA is usually monodentate (Amiraslanov *et al.*, 1978 ▸; Prondzinski & Merz, 2008 ▸), except in Cd^II^ complexes ABZCUH (Amiraslanov *et al.*, 1979*b* ▸) and BESRAS (Turner, *et al.*, 1982 ▸), where it is bidentate, and in RUPZIM where both mono- and bidentate coordination is present. Thus, compound (I) shows the most typical structural features, being a 1D coordination polymer with the *p*ABA bridge coordinated *via* the amino group and one carb­oxy­lic O atom (Fig. 2 ▸ *b*).

It is noteworthy that an isomer of the two-dimensional polymer IWORET (IWORET01; Crisan *et al.*, 2019 ▸) is one-dimensional and essentially isostructural with (I), with the same space group *P*2/*c* and similar unit-cell parameters, *a* = 7.0013 (4), *b* = 6.1301 (2), *c* = 17.1919 (7) Å, β = 92.148 (4)°, albeit without disorder. Another isomer of these, YIMDEO (Prondzinski & Merz, 2008 ▸) is 1D-polymeric, but with a tetra­hedral (O_3_N) metal coordination and different *p*ABA modes (Fig. 2 ▸ *a*,*b*).

## 7. Synthesis and crystallization

*Synthesis of (I)*. A mixture of Cu(NO_3_)_2_·2.5H_2_O (117 mg, 0.5 mmol), *p*ABAH (68.6 mg, 0.5 mmol) and 10 mL of H_2_O was placed in a 15 mL stainless steel-jacketed Teflon reactor. The reactor was carefully sealed, placed in the center of a programmable oven (Nabertherm 30–3000°C, S/N. 432847, 2022), and subjected to heating at a gradual rate of 0.1 K min^−1^ to 358 K, kept at the same temperature for a duration of 24 h, followed by gradual cooling of K min^−1^ to 298 K over 12 h. This afforded green block-shaped clear crystals. The obtained crystals were collected *via* filtration, washed with water (3 × 4 mL), then with ethanol (2 × 4 mL) and air-dried. Yield: 58 mg (65%), based on metal salt. Selected FTIR peaks (KBr, cm^−1^): 3250 (*br*), 3139 (*br*), 1606 (*s*), 1576 (*s*), 1304 (*s*), 1092 (*m*), 854 (*w*), 775 (*m*). The reaction synthesis is similar to that synthesized with H_3_bttta, except that 0.034 mmol (174 mg) of H_3_bttta were used instead of 0.5 mmol (34.8 mg) of *p*ABAH.

## 8. Refinement

Crystal data, data collection and structure refinement details are summarized in Table 2 ▸. The crystal studied was a merohedral twin with the twin components of equal size related by a 180° rotation about the *c* axis. The water H atoms were refined in isotropic approximation, other H atoms as riding in idealized positions, with *U*_iso_(H) = 1.2×*U*_eq_ of the bearing C or N atom.

## Supplementary Material

Crystal structure: contains datablock(s) I. DOI: 10.1107/S2056989024001336/zv2032sup1.cif

Structure factors: contains datablock(s) I. DOI: 10.1107/S2056989024001336/zv2032Isup2.hkl

CCDC reference: 2332153

Additional supporting information:  crystallographic information; 3D view; checkCIF report

## Figures and Tables

**Table 1:** Hydrogen-bond geometry (Å, °)

*D*—H⋯*A*	*D*—H	H⋯*A*	*D*⋯*A*	*D*—H⋯*A*
O3—H3⋯O2^i^	0.96 (1)	1.84 (1)	2.789 (3)	173 (2)
O4—H4⋯O2^i^	0.96 (1)	1.88 (2)	2.791 (3)	159 (3)
N—H1*A*⋯O2^ii^	0.91	2.26	2.954 (3)	133
N—H2*A*⋯O4^iii^	0.91	2.17	3.050 (4)	163
N—H1*B*⋯O2^ii^	0.91	2.26	2.954 (3)	133
N—H2*B*⋯O3^iv^	0.91	2.20	3.086 (4)	163

Symmetry codes: (i) 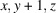 ; (ii) 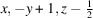 ; (iii) 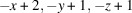 ; (iv) 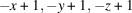 .

**Table 2:** Experimental details

Crystal data	
Chemical formula	[Cu(C_7_H_4_NO_2_)_2_(H_2_O)]·H_2_O
*M*_r_	371.83
Crystal system, space group	Monoclinic, *P*2/*c*
Temperature (K)	100
*a*, *b*, *c* (Å)	6.9143 (14), 6.2111 (12), 17.169 (3)
β (°)	90.05 (3)
*V* (Å^3^)	737.3 (3)
*Z*	2
Radiation type	Mo *K*α
μ (mm^−1^)	1.52
Crystal size (mm)	0.3 × 0.2 × 0.2

Data collection	
Diffractometer	Bruker SMART APEXII
Absorption correction	Multi-scan (*SADABS*; Krause *et al.*, 2015 ▸)
*T*_min_, *T*_max_	0.618, 0.745
No. of measured, independent and observed [*I* > 2σ(*I*)] reflections	8617, 1477, 1311
*R*_int_	0.027
(sin θ/λ)_max_ (Å^−1^)	0.642

Refinement	
*R*[*F* ^2^ > 2σ(*F* ^2^)], *wR*(*F* ^2^), *S*	0.033, 0.087, 1.09
No. of reflections	1477
No. of parameters	131
No. of restraints	4
H-atom treatment	H atoms treated by a mixture of independent and constrained refinement
Δρ_max_, Δρ_min_ (e Å^−3^)	0.37, −0.29

Computer programs: *APEX2* (Bruker, 2019 ▸), *SAINT-Plus* (Bruker, 2020 ▸), *SHELXT* (Sheldrick, 2015*a* ▸), *SHELXL* (Sheldrick, 2015*b* ▸), and *OLEX2* (Dolomanov *et al.*, 2009 ▸).

## supplementary crystallographic information

### Crystal data

**Table:**

[Cu(C_7_H_4_NO_2_)_2_(H_2_O)]·H_2_O	*F*(000) = 382
*M**_r_* = 371.83	*D*_x_ = 1.675 Mg m^−^^3^
Monoclinic, *P*2/*c*	Mo *K*α radiation, λ = 0.71073 Å
*a* = 6.9143 (14) Å	Cell parameters from 2372 reflections
*b* = 6.2111 (12) Å	θ = 3.0–26.0°
*c* = 17.169 (3) Å	µ = 1.52 mm^−^^1^
β = 90.05 (3)°	*T* = 100 K
*V* = 737.3 (3) Å^3^	Block, clear dark green
*Z* = 2	0.3 × 0.2 × 0.2 mm

### Data collection

**Table:**

Bruker SMART APEXII diffractometer	1477 independent reflections
Radiation source: sealed X-ray tube, EIGENMANN GmbH	1311 reflections with *I* > 2σ(*I*)
Graphite monochromator	*R*_int_ = 0.027
Detector resolution: 7.9 pixels mm^-1^	θ_max_ = 27.1°, θ_min_ = 1.2°
ω and φ scans	*h* = −8→8
Absorption correction: multi-scan (SADABS; Krause *et al.*, 2015)	*k* = −7→4
*T*_min_ = 0.618, *T*_max_ = 0.745	*l* = −20→21
8617 measured reflections	

### Refinement

**Table:**

Refinement on *F*^2^	Primary atom site location: dual
Least-squares matrix: full	Hydrogen site location: mixed
*R*[*F*^2^ > 2σ(*F*^2^)] = 0.033	H atoms treated by a mixture of independent and constrained refinement
*wR*(*F*^2^) = 0.087	*w* = 1/[σ^2^(*F*_o_^2^) + (0.0395*P*)^2^ + 0.5003*P*] where *P* = (*F*_o_^2^ + 2*F*_c_^2^)/3
*S* = 1.09	(Δ/σ)_max_ < 0.001
1477 reflections	Δρ_max_ = 0.37 e Å^−^^3^
131 parameters	Δρ_min_ = −0.29 e Å^−^^3^
4 restraints	

### Special details

**Table:**

Geometry. All esds (except the esd in the dihedral angle between two l.s. planes) are estimated using the full covariance matrix. The cell esds are taken into account individually in the estimation of esds in distances, angles and torsion angles; correlations between esds in cell parameters are only used when they are defined by crystal symmetry. An approximate (isotropic) treatment of cell esds is used for estimating esds involving l.s. planes.
Refinement. Refined as a 2-component twin.

### Fractional atomic coordinates and isotropic or equivalent isotropic displacement parameters (Å^2^)

**Table:**

	*x*	*y*	*z*	*U*_iso_*/*U*_eq_	Occ. (<1)
Cu1	0.500000	0.70928 (13)	0.750000	0.0206 (2)	0.6196 (16)
Cu2	1.000000	0.7095 (2)	0.750000	0.0204 (4)	0.3804 (16)
O1A	0.6443 (6)	0.6835 (6)	0.6512 (2)	0.0262 (8)	0.6196 (16)
O1B	0.8560 (9)	0.6813 (9)	0.6519 (4)	0.0243 (13)	0.3804 (16)
O2	0.7512 (5)	0.3523 (3)	0.68132 (10)	0.0363 (5)	
O3	0.500000	1.0567 (5)	0.750000	0.0468 (9)	
H3	0.586 (4)	1.1507 (11)	0.7225 (18)	0.070*	
O4	1.000000	1.0536 (5)	0.750000	0.0450 (9)	
H4	0.933 (5)	1.1481 (11)	0.7152 (15)	0.067*	
N	0.7528 (6)	0.3218 (3)	0.30662 (11)	0.0259 (5)	
H2A	0.820104	0.197454	0.299519	0.031*	0.6196 (16)
H1A	0.820685	0.428119	0.282487	0.031*	0.6196 (16)
H1B	0.684525	0.428327	0.282761	0.031*	0.3804 (16)
H2B	0.685060	0.197642	0.299792	0.031*	0.3804 (16)
C1A	0.7092 (8)	0.5003 (14)	0.6330 (5)	0.0224 (11)	0.6196 (16)
C1B	0.7949 (14)	0.492 (3)	0.6320 (9)	0.0224 (11)	0.3804 (16)
C2	0.7486 (7)	0.4541 (4)	0.54737 (13)	0.0284 (6)	
C3	0.7403 (8)	0.6201 (4)	0.49334 (15)	0.0424 (8)	
H3A	0.731206	0.764858	0.510783	0.051*	
C4	0.7451 (8)	0.5766 (4)	0.41429 (14)	0.0344 (7)	
H4A	0.742225	0.691677	0.377838	0.041*	
C5	0.7542 (8)	0.3680 (4)	0.38847 (13)	0.0268 (5)	
C6	0.7623 (8)	0.2011 (4)	0.44200 (17)	0.0494 (10)	
H6	0.768947	0.056284	0.424427	0.059*	
C7	0.7608 (8)	0.2452 (4)	0.52089 (16)	0.0420 (8)	
H7	0.768247	0.130184	0.557248	0.050*	

### Atomic displacement parameters (Å^2^)

**Table:**

	*U*^11^	*U*^22^	*U*^33^	*U*^12^	*U*^13^	*U*^23^
Cu1	0.0307 (4)	0.0221 (4)	0.0090 (3)	0.000	−0.0030 (12)	0.000
Cu2	0.0274 (7)	0.0220 (6)	0.0119 (6)	0.000	−0.005 (2)	0.000
O1A	0.038 (2)	0.031 (2)	0.0097 (17)	0.0077 (18)	0.0008 (19)	0.0004 (17)
O1B	0.037 (3)	0.016 (3)	0.020 (3)	−0.005 (3)	−0.002 (4)	0.000 (3)
O2	0.0657 (13)	0.0277 (8)	0.0155 (8)	0.0000 (13)	0.001 (2)	0.0047 (7)
O3	0.0460 (19)	0.0244 (16)	0.070 (2)	0.000	0.017 (4)	0.000
O4	0.0417 (18)	0.0358 (18)	0.057 (2)	0.000	−0.017 (4)	0.000
N	0.0398 (13)	0.0253 (9)	0.0127 (9)	0.0015 (17)	−0.001 (2)	−0.0010 (8)
C1A	0.027 (3)	0.0214 (14)	0.0188 (14)	0.002 (4)	0.002 (4)	−0.0026 (11)
C1B	0.027 (3)	0.0214 (14)	0.0188 (14)	0.002 (4)	0.002 (4)	−0.0026 (11)
C2	0.0508 (16)	0.0221 (12)	0.0121 (11)	0.002 (2)	0.004 (3)	−0.0001 (9)
C3	0.093 (3)	0.0178 (12)	0.0171 (12)	−0.006 (3)	−0.002 (3)	−0.0025 (10)
C4	0.066 (2)	0.0218 (11)	0.0155 (11)	0.000 (2)	0.003 (3)	0.0031 (9)
C5	0.0412 (14)	0.0268 (12)	0.0123 (10)	0.002 (2)	0.000 (2)	−0.0026 (9)
C6	0.107 (3)	0.0222 (13)	0.0189 (13)	0.008 (3)	0.000 (3)	−0.0037 (10)
C7	0.090 (3)	0.0196 (11)	0.0167 (13)	0.004 (2)	−0.001 (2)	0.0046 (10)

### Geometric parameters (Å, º)

**Table:**

Cu1—O1A^i^	1.975 (4)	N—H2A	0.9100
Cu1—O1A	1.975 (4)	N—H1A	0.9100
Cu1—O3	2.158 (3)	N—H1B	0.9100
Cu1—N^ii^	2.009 (4)	N—H2B	0.9100
Cu1—N^iii^	2.009 (4)	N—C5	1.434 (3)
Cu2—O1B	1.964 (6)	C1A—C2	1.523 (9)
Cu2—O1B^iv^	1.964 (6)	C1B—C2	1.506 (16)
Cu2—O4	2.137 (3)	C2—C3	1.388 (3)
Cu2—N^ii^	1.976 (4)	C2—C7	1.377 (3)
Cu2—N^v^	1.976 (4)	C3—H3A	0.9500
O1A—C1A	1.262 (9)	C3—C4	1.384 (3)
O1B—C1B	1.294 (17)	C4—H4A	0.9500
O2—C1A	1.272 (9)	C4—C5	1.371 (3)
O2—C1B	1.251 (16)	C5—C6	1.386 (4)
O3—H3^i^	0.957 (3)	C6—H6	0.9500
O3—H3	0.957 (3)	C6—C7	1.382 (4)
O4—H4	0.958 (3)	C7—H7	0.9500
O4—H4^iv^	0.958 (3)		
			
O1A^i^—Cu1—O1A	170.7 (2)	C5—N—Cu2^v^	119.8 (3)
O1A—Cu1—O3	94.66 (12)	C5—N—H2A	107.4
O1A^i^—Cu1—O3	94.66 (12)	C5—N—H1A	107.4
O1A^i^—Cu1—N^ii^	90.96 (15)	C5—N—H1B	107.4
O1A^i^—Cu1—N^iii^	88.14 (15)	C5—N—H2B	107.4
O1A—Cu1—N^iii^	90.96 (15)	O1A—C1A—O2	124.8 (7)
O1A—Cu1—N^ii^	88.14 (15)	O1A—C1A—C2	118.3 (7)
N^iii^—Cu1—O3	95.52 (6)	O2—C1A—C2	116.9 (6)
N^ii^—Cu1—O3	95.52 (6)	O1B—C1B—C2	117.8 (12)
N^iii^—Cu1—N^ii^	168.96 (12)	O2—C1B—O1B	122.1 (13)
O1B^iv^—Cu2—O1B	169.8 (3)	O2—C1B—C2	119.5 (11)
O1B—Cu2—O4	95.11 (17)	C3—C2—C1A	119.8 (4)
O1B^iv^—Cu2—O4	95.11 (17)	C3—C2—C1B	122.4 (7)
O1B^iv^—Cu2—N^ii^	90.4 (2)	C7—C2—C1A	120.5 (4)
O1B—Cu2—N^ii^	88.6 (2)	C7—C2—C1B	117.0 (6)
N^ii^—Cu2—O4	95.64 (7)	C7—C2—C3	118.8 (2)
N^v^—Cu2—O4	95.64 (7)	C2—C3—H3A	119.7
N^ii^—Cu2—N^v^	168.72 (13)	C4—C3—C2	120.6 (2)
C1A—O1A—Cu1	117.8 (5)	C4—C3—H3A	119.7
C1B—O1B—Cu2	118.2 (8)	C3—C4—H4A	119.9
Cu1—O3—H3^i^	127.6 (4)	C5—C4—C3	120.2 (2)
Cu1—O3—H3	127.6 (4)	C5—C4—H4A	119.9
H3—O3—H3^i^	104.8 (8)	C4—C5—N	120.4 (2)
Cu2—O4—H4	127.8 (4)	C4—C5—C6	119.6 (2)
H4—O4—H4^iv^	104.4 (8)	C6—C5—N	120.0 (2)
Cu1^iii^—N—H2A	107.4	C5—C6—H6	120.0
Cu1^iii^—N—H1A	107.4	C7—C6—C5	120.1 (2)
Cu2^v^—N—H1B	107.4	C7—C6—H6	120.0
Cu2^v^—N—H2B	107.4	C2—C7—C6	120.7 (2)
H2A—N—H1A	106.9	C2—C7—H7	119.6
H1B—N—H2B	106.9	C6—C7—H7	119.6
C5—N—Cu1^iii^	119.9 (3)		
			
Cu1—O1A—C1A—O2	−26.2 (8)	O2—C1B—C2—C3	161.0 (7)
Cu1—O1A—C1A—C2	156.5 (4)	O2—C1B—C2—C7	−34.5 (11)
Cu1^iii^—N—C5—C4	−86.6 (6)	N—C5—C6—C7	−179.1 (5)
Cu1^iii^—N—C5—C6	92.6 (5)	C1A—C2—C3—C4	−169.7 (5)
Cu2—O1B—C1B—O2	30.0 (12)	C1A—C2—C7—C6	168.5 (5)
Cu2—O1B—C1B—C2	−159.1 (6)	C1B—C2—C3—C4	163.9 (6)
Cu2^v^—N—C5—C4	93.1 (5)	C1B—C2—C7—C6	−165.9 (7)
Cu2^v^—N—C5—C6	−87.7 (5)	C2—C3—C4—C5	1.4 (9)
O1A—C1A—C2—C3	9.4 (9)	C3—C2—C7—C6	−0.8 (8)
O1A—C1A—C2—C7	−159.8 (5)	C3—C4—C5—N	178.0 (5)
O1B—C1B—C2—C3	−10.2 (12)	C3—C4—C5—C6	−1.3 (9)
O1B—C1B—C2—C7	154.3 (7)	C4—C5—C6—C7	0.2 (9)
O2—C1A—C2—C3	−168.2 (5)	C5—C6—C7—C2	0.9 (9)
O2—C1A—C2—C7	22.7 (8)	C7—C2—C3—C4	−0.3 (8)

Symmetry codes: (i) −*x*+1, *y*, −*z*+3/2; (ii) *x*, −*y*+1, *z*+1/2; (iii) −*x*+1, −*y*+1, −*z*+1; (iv) −*x*+2, *y*, −*z*+3/2; (v) −*x*+2, −*y*+1, −*z*+1.

### Hydrogen-bond geometry (Å, º)

**Table:**

*D*—H···*A*	*D*—H	H···*A*	*D*···*A*	*D*—H···*A*
O3—H3···O2^vi^	0.96 (1)	1.84 (1)	2.789 (3)	173 (2)
O4—H4···O2^vi^	0.96 (1)	1.88 (2)	2.791 (3)	159 (3)
N—H1*A*···O2^vii^	0.91	2.26	2.954 (3)	133
N—H2*A*···O4^v^	0.91	2.17	3.050 (4)	163
N—H1*B*···O2^vii^	0.91	2.26	2.954 (3)	133
N—H2*B*···O3^iii^	0.91	2.20	3.086 (4)	163

Symmetry codes: (iii) −*x*+1, −*y*+1, −*z*+1; (v) −*x*+2, −*y*+1, −*z*+1; (vi) *x*, *y*+1, *z*; (vii) *x*, −*y*+1, *z*−1/2.
